# Blood–Brain Barrier Function and Biomarkers of Central Nervous System Injury in Rickettsial versus Other Neurological Infections in Laos

**DOI:** 10.4269/ajtmh.15-0119

**Published:** 2015-08-05

**Authors:** Sabine Dittrich, Piyanate Sunyakumthorn, Sayaphet Rattanavong, Rattanaphone Phetsouvanh, Phonepasith Panyanivong, Amphonsavanh Sengduangphachanh, Phonelavanh Phouminh, Tippawan Anantatat, Anisone Chanthongthip, Sue J. Lee, Audrey Dubot-Pérès, Nicholas P. J. Day, Daniel H. Paris, Paul N. Newton, Gareth D. H. Turner

**Affiliations:** Microbiology Laboratory, Lao-Oxford-Mahosot Hospital Wellcome Trust Research Unit (LOMWRU), Mahosot Hospital, Vientiane, Lao People's Democratic Republic; Nuffield Department of Clinical Medicine, Centre for Tropical Medicine and Global Health, University of Oxford, Oxford, United Kingdom; Mahidol-Oxford Tropical Medicine Research Unit (MORU), Faculty of Tropical Medicine, Mahidol University, Bangkok, Thailand; IRD French Institute of Research for Development, UMR 190 “Emergence des Pathologies Virales,” Aix-Marseille University, EHESP French School of Public Health, Marseilles, France

## Abstract

Blood–brain barrier (BBB) function and cerebrospinal fluid (CSF) biomarkers were measured in patients admitted to hospital with severe neurological infections in the Lao People's Democratic Republic (*N* = 66), including bacterial meningitis (BM; *N* = 9) or tuberculosis meningitis (TBM; *N* = 11), Japanese encephalitis virus (JEV; *N* = 25), and rickettsial infections (*N* = 21) including murine and scrub typhus patients. The albumin index (AI) and glial fibrillary acidic protein (GFAP) levels were significantly higher in BM and TBM than other diseases but were also raised in individual rickettsial patients. Total tau protein was significantly raised in the CSF of JEV patients. No differences were found between clinical or neurological symptoms, AI, or biomarker levels that allowed distinction between severe neurological involvement by *Orientia tsutsugamushi* compared with *Rickettsia* species.

Central nervous system (CNS) infections are caused by a range of different pathogens and a major cause of morbidity and mortality worldwide.^1^,^2^ *Orientia tsutsugamushi* and *Rickettsia* spp. infections have recently been identified as a major cause of CNS disease in Lao People's Democratic Republic (Laos) in a large prospective study^3^ where 9% of all CNS infections were caused by *O. tsutsugamushi*, *Rickettsia* spp., or *Leptospira* spp.^4^ Differentiating these organisms in scrub and murine typhus patients from other causes of meningoencephalitis such as bacterial or tuberculous meningitis is diagnostically challenging. Neurological manifestations of severe typhus occur in up to 10% of cases, with headache, photophobia and meningeal symptoms, decreased consciousness, or even death.^4^–^8^ Japanese encephalitis virus (JEV) is also an important cause of CNS disease.^9^ Neuropathological data from autopsy cases of rickettsial and JEV deaths are limited,^10^ so studying the cerebrospinal fluid (CSF) of living patients may help diagnosis and our understanding of the pathophysiology of CNS rickettsial, as opposed to other, infections.^11^,^12^

This study compared blood–brain barrier (BBB) function and CSF biomarkers of cellular activation and injury in patients with severe neurological infections from Laos and explored their relationship with clinical presentation and laboratory findings. Patients (*N* = 66) were part of a hospital-based prospective study of CNS infections and included if matching samples of admission plasma and CSF were available. Ethical approval was granted by OXTREC (015-02, University of Oxford, United Kingdom) and the Faculty of Medical Sciences Committee (University of Health Sciences, Lao PDR).^3^ The following groups were included: bacterial meningitis (BM: *N* = 9; *Streptococcus pneumonia* [*N* = 5], *Neisseria meningitides* [*N* = 2], *S. suis* [*N* = 1], *S. viridans* [*N* = 1]); *Mycobacterium tuberculosis* meningitis (TBM, *N* = 11); Japanese B encephalitis virus (JEV, *N* = 25), and rickettsial infections (*N* = 21): *O. tsutsugamushi* (*N* = 11), *Rickettsia typhi* (*N* = 7), and (*N* = 3) other *Rickettsia* spp. Bacterial molecular diagnostics and *Rickettsia* culture and typing were performed as described.^3^ TBM was defined as CSF culture positivity for *M. tuberculosis* on Lowenstein–Jensen medium with subsequent molecular confirmation (GenoType MTBDRplus version 2; Hain Lifescience, Nehren, Germany). JEV cases were confirmed by enzyme-linked immunosorbent assay (ELISA) on CSF using the Japanese encephalitis Dengue IgM Combo ELISA test (E-JED01C, Panbio, Japan) or by pan-flavivirus polymerase chain reaction (PCR) and sequencing (*N* = 1; Macrogene, Korea; NCBI/Blastn: Identity 97% to GQ902059.1, E-value: 8e-79, coverage: 100%, c782-PF3PF2b:GGTTCATGTGGCTGGGAGCACGGTACCTAGAGTTTGAAGCCCTAGGATTTCTAAATGAAGACCATTGGCTGAGCCGAGAGAATTCAGGAGGCGGGGTGGAAGGTTCAGGCGTCCAAAAGCTGGGATACATCCTCCGTGACATTGCAGGGAAGCAAGGAGGAAAAATGTATGCCGATGA).^13^ Changes in BBB function were assessed using the albumin index (AI; [AI = (CSF/plasma albumin)× 10^3^]) to determine leakage across the BBB.^9^ Plasma and CSF (1:2,000 dilution) were tested using human plasma albumin ELISA (Assaypro, St. Charles, MO) and CSF albumin ELISA kits (Abnova, Taipei City, Taiwan), respectively. Other biomarkers were measured using commercial ELISA kits according to manufacturer's instructions to assess the astrocyte marker glial fibrillary acidic protein (GFAP; BioVendor, Brno, Czech Republic) and S100b for astroglial cells (BioVendor), neuron-specific enolase (NSE; USCN, Hubei, China), and total tau protein for axonal/neuronal damage (Invitrogen, Carlsbad, CA).^12^ CSF samples were used undiluted (S100b, GFAP) or diluted (tau 1:2; NSE 1:10). Optical density values at 450 nm were determined by spectrophotometer (Multiskan Go; Thermo scientific, Waltham, MA), and the albumin/biomarker concentration was calculated from standard curves. Data were summarized using medians (interquartile range, [IQR]) or frequencies (%). Pairwise associations between AI and biomarker levels with demographics, clinical signs and symptoms, severity and outcome measures, and laboratory measures were assessed using Kendall's rank correlation coefficient for continuous variables and the Mann–Whitney *U* test for categorical variables. Statistical analyses were done using Stata v12.0 (StataCorp LP, College Station, TX).

The demographics, clinical, and laboratory findings are summarized in Table 1. Clinical features of neurological rickettsial infection included relatively common incidence of seizures (7/21 = 33% for rickettsial infections, significantly higher than in BM or TBM, *P* = 0.003, commonest in JEV 12/25 = 48%), but no difference between scrub typhus versus other rickettsial infections (*P* = 0.663). There was a significant difference in mortality between groups with 7/11 (88%) of the TBM group dying, whereas 3/18 (17%) of rickettsial infected cases died. TBM mortality rates in this study were higher than in the larger clinical study (∼50%).^3^

BBB leakage, measured using AI, increased in all clinical groups (Figure 1A), although only TBM cases showed a significantly raised AI compared with the lowest group, JEV infection (*P* = 0.0081; Figure 1A

**Figure 1.**
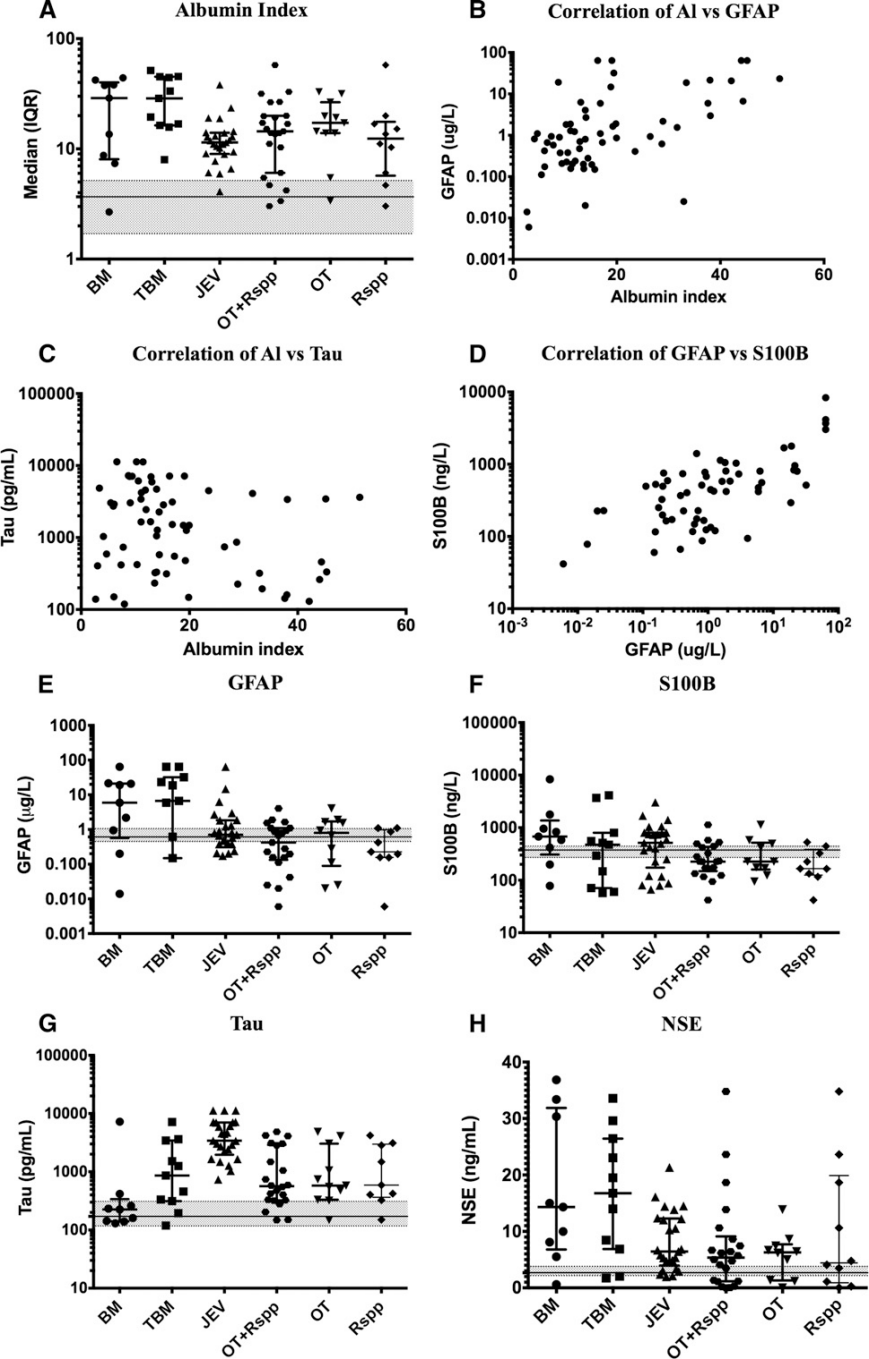
AI as a measure of blood–brain barrier (BBB) function, the correlation between AI and CSF biomarkers and levels of biomarkers of neurological injury, comparing different types of CNS infection. Middle lines indicate median; error bars represent IQR. (**A**) AI in different clinical groups. Shaded areas show control values of AI.^11^,^14^–^16^ (**B**) The correlation between AI and GFAP. (**C**) The correlation between AI and tau. (**D**) The correlation between GFAP and S100B. (**E**–**H**) Distribution of biomarkers in the CSF of individual patients shown as dot plots. The middle line indicating median, and error bars represent IQR. Shaded areas show control values.^15^,^16^ (**E**) GFAP (reference range: median = 0.61, IQR = 0.45–1.06), (**F**) S100B (reference range: median = 375, IQR = 270.4–443.5), (**G**) tau (reference range: median = 171, IQR = 117–310), and (**H**) NSE (reference range: median = 2.67, IQR = 2.17–3.80). AI = albumin index; BM = bacterial meningitis; CNS = central nervous system; CSF = cerebrospinal fluid; GFAP = glial fibrillary acidic protein; IQR = interquartile range; JEV = Japanese encephalitis virus; NSE = neuron-specific enolase; OT = *Orientia tsutsugamushi*; Rspp = *Rickettsia* genus; TBM = *Mycobacterium tuberculosis* meningitis.

). Patients with scrub typhus (median = 17.2, IQR = 13.9–26.5) showed a nonsignificantly higher AI than other rickettsial infections (median = 12.4, IQR = 6.1–16.8). Individual cases with both infections showed markedly raised AI, significantly correlated with higher levels of CSF lactate, white cell counts, and protein, but not CSF opening pressure (Table 2).

Tau and GFAP are only produced in the brain, and raised AI was significantly correlated with GFAP levels (*P* = 0.0001; Figure 1B), but not tau (*P* = 0.043; Figure 1C). NSE can be produced elsewhere in the body, so increased levels in the CSF could reflect leak across the BBB from the blood. NSE levels were positively correlated with AI as a marker for BBB leakage (*P* < 0.0001). Both GFAP and S100b are markers of astrocytic activation, and raised levels reflect either activation or damage to the BBB. A strong correlation between GFAP and S100b levels was seen (rho = 0.489, *P* < 0.0001; Figure 1D). GFAP was highest in TBM and BM cases but not significantly different across groups (*P* = 0.0678). Rickettsial patients showed GFAP and S100b levels generally within or around normal range compared with TBM and BM cases (Figure 1E–F). Total tau was significantly higher in the JEV group compared with other groups (*P* = 0.0001, Figure 1G), with rickettsial infections showing higher median levels than BM cases, suggestive of neuronal/axonal damage (Table 1). NSE levels varied widely but were highest in BM and TBM cases, but not significantly different between the disease groups (Figure 1H and Table 1).

Observed BBB function measured by AI was independent of admission weight or hematocrit, and no relationship was found between AI and the Glasgow Coma Scale (GCS) score (Table 2). CSF lactate was also significantly higher in TBM and BM (*P* = 0.001) than other groups. A significant correlation between AI and CSF/blood glucose ratio (*P* = 0.0001) was found. Levels of tau were significantly higher in patients with lower GCS (*P* = 0.0095) and borderline significant for S100b (*P* = 0.0237), NSE (*P* = 0.0213), and GFAP (*P* = 0.0107; Table 2), implying higher CSF biomarker levels in cases with more severe neurological injury and deeper coma score.

There were no differences between the scrub typhus group infected with *O. tsutsugamushi* versus *Rickettsia* spp. (including patients with murine typhus) either in AI (*P* = 0.51) or the levels of other biomarkers (GFAP: *P* = 0.48, S100b: *P* = 0.29, NSE: *P* = 0.79, and tau: *P* = 0.62). No significant differences were found between demographic data, outcome, CSF lactate, and protein or glucose levels between scrub typhus and other rickettsial infections.

All clinical groups showed raised AI compared with normal levels, although individual cases were within normal range. This study could not compare control CSF from uninfected patients (for ethical reasons), so normal control ranges from previous studies were used.^11^,^14^–^16^ The degree of BBB leakage varied between and within groups, similar to a previous study of neurological infections in Vietnam.^11^ TBM and BM cases had significantly higher BBB leakage and more obvious inflammatory responses in the CSF with raised lactate, leukocytosis, protein release, and decreased CSF/blood glucose ratio, compared with JEV or rickettsial infections. Both scrub and murine typhus patients showed heterogeneous results, with individual patients showing very high BBB leakage, but overall not significantly different from other causes of neurological infection.

Changes in BBB function were strongly correlated with rises in both GFAP and NSE. GFAP levels were higher in diseases also showing BBB leakage, including BM and TBM. This is consistent with a primary function of astrocytes in maintaining structural integrity of the BBB, so increased AI is reflected in higher astrocyte markers. NSE is released in chronic and acute neuronal damage, for instance after seizures, but no significant difference was seen in NSE levels between groups.

A novel finding of this study was the significant rise in the neuronal/axonal marker (total) tau in the group with JEV. Tau is a phosphoprotein that binds tubulin and promotes microtubule assembly and stability. Raised levels reflect rapidly progressive neuroaxonal degeneration, as reported in dementia and multiple sclerosis.^16^ Although raised tau in JEV cases indicates acute release, as might be expected in a neurotropic virus, the lack of raised NSE in the same cases argues for a process affecting axons rather than neurons. Further diagnostic studies using tau and other axonal injury markers such as amyloid precursor protein (beta APP) are required in larger cohorts of JEV patients.

This study aimed to examine BBB and CSF biomarkers as aids to the diagnosis and understanding of CNS rickettsial disease, in comparison to other severe neurological infections. CSF examination alone, or addition of biomarkers, could not differentiate rickettsial from other neurological infections in this setting. No significant differences could be found between either rickettsial patients compared with other groups or between scrub and murine typhus patients. The results indicate that microbiological investigation remains the mainstay of diagnosis to guide treatment, as adjuvant biomarkers were not helpful given the heterogeneous host response to neurological rickettsial infection.

## Figures and Tables

**Table 1:** Demographic and clinical details, laboratory findings, AI, and biomarker levels by disease group

	All patients	BM	TBM	JEV	*Rickettsia* spp.	*Orientia tsutsugamushi*	Grouped *Rickettsia* pathogens
	*N* = 66	*N* = 9	*N* = 11	*N* = 25	*N* = 10	*N* = 11	*N* = 21
Demographic and general data							
Age (years)	19 (13–38)	43 (18–53)	35 (22–52)	16 (8–20)	27 (16–48)	16 (7–35)	19 (13–41)
Age < 15 years	18 (27.3)	0 (0)	0 (0)	12 (48.0)	2 (20)	4 (36.4)	6 (28.6)
Weight (kg)	45 (20–54); 23	54 (43–55); 2	50 (47–55); 5	27 (14–45); 7	48 (34–50); 4	35 (16.5–55); 5	48 (18.3–52.5); 9
Male	46 (69.7)	7 (77.8)	8 (72.7)	18 (72.0)	6 (60.0)	7 (63.6)	13 (61.9)
Clinical signs and symptoms							
Temperature at admission (°C)	38.0 (37.5–39.0)	37.5 (37.5–38.0)	38.0 (37.7–39.3)	38.0 (37.0–39.0)	38.0 (36.6–38.5)	38.5 (37.5–39.5)	38.5 (37.5–38.5)
Headache	57 (86.4)	9 (100)	10 (90.9)	22 (88.0)	9 (90.0)	7 (63.6)	16 (76.2)
Vomiting	38 (57.6)	4 (44.4)	5 (45.5)	17 (68.0)	5 (50.0)	7 (63.6)	12 (57.1)
Seizure	22 (33.3)	0 (0)	1 (9.09)	12 (56.0)	3 (30.0)	4 (36.4)	7 (33.3)
Stiff neck	47 (71.2)	6 (66.7)	7 (63.6)	21 (84.0)	6 (60.0)	7 (63.6)	13 (61.9)
Skin rash	6 (9.09)	0 (0)	2 (18.2)	2 (8.00)	0 (0)	2 (18.2)	2 (9.52)
Hearing loss	4 (6.06)	1 (11.1)	0 (0)	2 (12.0)	0 (0)	0 (0)	0 (0)
Photophobia	1 (1.52)	0 (0)	0 (0)	0 (0)	0 (0)	1 (9.09)	1 (4.76)
Visual loss	7 (10.6)	2 (22.1)	1 (9.1)	3 (12.0)	1 (10)	0 (0)	1 (4.8)
Eschar	1 (1.52)	0 (0)	0 (0)	0 (0)	0 (0)	1 (9.09)	1 (4.76)
Severity and outcome measures							
Died	13 (25.5); 15	1 (14.3); 2	7 (87.5); 3	2 (11.1); 7	1 (11.1); 1	2 (22.2); 2	3 (16.7); 3
GCS	13 (10–15); 1	14 (11–15)	12 (7–15)	12 (9–14); 1	13.5 (10–15)	15 (13–15)	14 (12–15)
GCS < 15	45 (69.2); 1	6 (66.7)	8 (72.7)	19 (79.2); 1	7 (70.0)	5 (45.5)	12 (57.1)
Meningism (WHO)	52 (78.8)	6 (66.7)	8 (72.7)	22 (88.0)	7 (70.0)	9 (81.8)	16 (76.2)
AES (WHO)	50 (75.8)	5 (55.6)	8 (72.7)	23 (92.0)	7 (70.0)	7 (63.6)	14 (66.7)
Meningism and AES (WHO)	47 (71.2)	5 (55.6)	7 (63.6)	22 (88.0)	7 (70.0)	6 (54.6)	13 (61.9)
Laboratory investigation							
Opening pressure (cm H_2_O)*	20 (15–34); 2	19.0 (10.3–34.3); 1	36.5 (17.0–41.0); 1	21.0 (17.0–27.0)	15.8 (12.5–20.0)	20.0 (16.0–40.0)	20.0 (15.0–34.0)
Turbid	10 (16.1); 4	4 (44.4)	1 (9.09)	2 (8.33); 1	1 (12.5); 2	2 (20.0); 1	3 (16.7)
Total white cell count/mm^3^	88 (10–286)	286 (45–1,085)	170 (85–385)	80 (20–225)	8 (0–25)	115 (5–275)	25 (0–165)
Neutrophils ≥ 1/mm^3^	53 (80.3)	7 (77.8)	10 (90.9)	22 (88.0)	5 (50.0)	9 (81.8)	14 (66.7)
Median (range)	40.3 (5.00–130)	280 (30.0–738)	95.0 (30.0–165)	35.2 (5.00–110)	5.00 (0–25.0)	44.8 (5.00–130)	10.0 (0–65.0.)
Lymphocytes > 5/mm^3^	42 (63.6)	6 (66.7)	9 (81.8)	18 (72.0)	3 (30.0)	6 (54.6)	9 (42.9)
Median (range)	21.1 (3.90–75.0)	20 (4.95–64.0)	55.0 (30.0–245)	24.8 (5.00–115)	0 (0–15.0)	20.8 (0–100)	5.00 (0–25.2)
Neutrophil:lymphocyte ratio	1.27 (0.37–3.20); 16	9.0 (2.13–49.0); 2	2.55 (0.55–3.17); 1	0.70 (0.52–3.00); 3	0.43 (0.10–1.00); 6	1.30 (0.15–6.69); 4	1.00 (0.15–1.78); 10
Lactate > 4 mmol/L	19 (31.7); 6	4 (50.0); 1	8 (80.0); 1	2 (8.33); 1	2 (25.0); 2	3 (30.0); 1	5 (27.8); 3
Glucose < 2.5 mmol/L	14 (22.2); 3	1 (12.5); 1	4 (36.4)	7 (28.0)	0 (0); 2	2 (18.2)	2 (10.5); 2
Protein > 40 mg/L	44 (71.0); 4	6 (85.7); 2	8 (72.7)	17 (68.0)	4 (50.0); 2	9 (81.8)	13 (68.4); 2
CSF/blood glucose ratio < 0.5	40 (69.0); 8	2 (50.0); 5	11 (100)	14 (58.3); 1	5 (62.5); 2	8 (72.7)	13 (68.4); 2
Biomarkers							
Plasma albumin (g/L)	34.0 (26.8–39.3)	36.2 (32.9–44.3)	29.7 (20.2–35.6)	34.7 (28.8–40.8)	39.5 (30.6–44.7)	33.2 (22.0–35.1)	34.2 (25.4–39.3)
CSF albumin (g/L)	0.44 (0.30–0.70)	1.00 (0.33–1.42)	0.74 (0.33–1.32)	0.37 (0.26–0.51)	0.42 (0.24–0.51)	0.56 (0.30–0.70)	0.47 (0.30–0.68)
AI = CSF:plasma/albumin ratio (×1,000)	13.9 (9.41–23.5)	28.9 (8.72–40.0)	28.7 (16.3–45.2)	11.4 (9.06–13.9)	12.4 (6.05–16.8)	17.2 (13.9–26.5)	14.4 (10.3–19.8)
CSF tau (pg/mL)	1,479 (404–4,103); 1	226 (143–261)	862 (313–3,439)	3,411 (2,268–6,997)	590 (404–2,845); 1	578 (331–3,032)	584 (368–2,939); 1
CSF GFAP (ug/L)	0.84 (0.21–3.50); 2	5.92 (0.94–20.7)	6.67 (0.15–32.0)	0.70 (0.38–1.82)	0.23 (0.16–0.86); 1	0.80 (0.11–1.64); 1	0.42 (0.16–1.10); 2
CSF S100b (ng/L)	421 (156–762); 2	677 (417–955)	473 (71–797)	513 (227–801)	166 (134–325); 1	226 (171–495); 1	225 (134–444); 2
CSF NSE (ng/mL)	6.88 (4.08–14.4); 1	14.3 (8.09–30.3)	16.8 (6.88–26.4)	6.43 (4.53–12.2)	4.41 (1.10–18.7)	6.29 (1.35–7.43); 1	5.59 (1.26–9.62); 1

AES = acute encephalitis syndrome; AI = albumin index; BM = bacterial meningitis; CSF = cerebrospinal fluid; GFAP = glial fibrillary acidic protein; IQR = interquartile range; JEV = Japanese encephalitis virus; NSE = neuron-specific enolase; TBM = *Mycobacterium tuberculosis* meningitis; WHO = World Health Organization.

Grouped *Rickettsia* pathogens: *Rickettsia* spp. and *O. tsutsugamushi*. Data are given as “median (IQR); missing” or “number (%); missing.” “AES” was defined according to WHO guidelines “as a person of any age, at any time of year with the acute onset of fever and either a change in mental status (including symptoms such as confusion, disorientation, coma, or inability to talk) and/or new onset of seizures (excluding simple febrile seizures).” “Meningitis” was defined according to WHO guidelines as “a sudden onset of fever (> 38.5°C rectal or 38.0°C axillary) with one of the following signs: neck stiffness, altered consciousness, or other meningeal sign(s).” Meningoencephalitis was defined as fulfilling both criteria.

* 41 indicating > 40 cm H_2_O.

**Table 2:** Statistical comparisons of CSF results, clinical and laboratory data

	(log) AI	Tau	GFAP	S100b	NSE
Demography and general data					
Age	0.0215	0.0148	NS	NS	NS
Weight	NS	NS	NS	NS	NS
Clinical signs and symptoms					
Headache	NS	NS	NS	NS	NS
Vomiting	NS	NS	NS	NS	NS
Seizures	**0.0055**	**0.0003**	NS	NS	NS
Rash	NS	NS	NS	NS	NS
Hearing loss	NS	**0.0075**	NS	NS	NS
Photophobia	NS	NS	NS	NS	NS
Eschar	NS	NS	NS	NS	NS
Visual loss	NS	0.0422	NS	NS	NS
Severity and outcome measures					
Outcome	NS	NS	NS	NS	NS
GCS	NS	**0.0095**	0.0107	0.0237	0.0213
WHO meningism	NS	0.0134	0.0272	**0.0064**	0.0427
WHO AES	NS	**0.0001**	0.0312	0.0126	NS
WHO men and AES	NS	**0.0007**	NS	**0.0087**	NS
Laboratory investigations					
CSF opening pressure	NS	NS	NS	NS	NS
Turbidity	NS	NS	NS	NS	NS
CSF white cells/mm^3^	**< 0.0001**	NS	**0.0038**	**0.0024**	**0.0030**
CSF neutrophils/mm^3^	**0.0001**	NS	**0.0012**	**0.0017**	**0.0036**
CSF lymphocytes/mm^3^	**0.0004**	NS	NS	0.0337	0.0255
Blood/CSF glucose ratio	**0.0001**	NS	NS	NS	0.0437
CSF lactate > 4 mmol/L	**0.0001**	NS	**0.0001**	0.0446	**0.0143**
CSF glucose < 2.5 mmol/L	NS	NS	NS	NS	NS
CSF protein > 40 mg/L	**0.0020**	NS	NS	0.0481	0.0303
Bilirubin	NS	NS	NS	NS	NS
Hematocrit	NS	NS	NS	NS	NS

AES = acute encephalitic syndrome; AI = albumin index; CSF = cerebrospinal fluid; GFAP = glial fibrillary acidic protein; NS = nonsignificant; NSE = neuron-specific enolase; WHO = World Health Organization.

Comparisons across clinical groups were made using the Kruskal–Wallis equality-of-populations rank test. Because of the exploratory nature of this study and multiple comparisons, a conservative *P* value of < 0.01 was considered significant (shown in bold). Exact *P* values are reported (for values < 0.05) for Bonferroni correction (α/*n*, where α = 0.05 and *n* = number of tests), if preferred.
